# A Comprehensive Narrative Review of Nanomaterial Applications in Restorative Dentistry: Reinforcement and Therapeutic Applications (Part II)

**DOI:** 10.7759/cureus.80127

**Published:** 2025-03-06

**Authors:** Sahar M Elmarsafy

**Affiliations:** 1 Department of Restorative Dentistry, Faculty of Dental Medicine, Umm Al-Qura University, Makkah, SAU

**Keywords:** composite resin, dental restorations, drug delivery, nanodentistry, nanoparticles, nanorobotics, nanotechnology, nanotissue engineering, reinforcement, tooth regeneration

## Abstract

Nanotechnology has been widely introduced into many areas of dentistry, including restorative dentistry, where it has contributed greatly to the improvement of restorative materials and procedures. This review was conceived with the aim of exploring the various applications of nanotechnology in restorative dentistry. The review consists of two parts. The first part addressed applications for remineralization inhibition and remineralization. This current review is the second part aimed at focusing on the reinforcement of restorative materials and other therapeutic applications of nanomaterials. Among the nanoparticles that are used to reinforce restorative materials are carbon, zirconia, hydroxyapatite, titanium dioxide, alumina, and gold nanoparticles. Furthermore, other promising applications of nanotechnology are for hypersensitivity management, protective varnish, whitening effect, drug delivery, and nanorobotics, which includes performing major tooth repairs and conducting dentition renaturalization procedures. These applications highlight the potential of nanoparticles in restorative dentistry; however, there are still certain limitations that need to be handled.

## Introduction and background

Nanotechnology emerged in the 1950s and has since been integrated into numerous industrial and medicinal sectors, achieving significant breakthroughs. The fundamental concept of its uniqueness lies in the potential to create nanomaterials that are lighter yet stronger, exhibiting superior physical, mechanical, and biological capabilities [1]. Nano denotes one billionth of a meter or a factor of 10^−9^, and to enhance understanding, a nanoscale was established that ranges from 1 to 100 nm. Nanomaterials are categorized based on their structure when at least one of their dimensions is under 100 nm. A further, deeper classification exists based on its structural properties [1,2]. Nanotechnology has spread to various domains, particularly dentistry, with a significant impact on restorative dentistry; it enhances the clinical efficacy of restorative materials by improving their antibacterial properties, inhibiting mineral loss, promoting remineralization, augmenting mechanical properties, and establishing broad treatment modalities [3,4]. Although there are many reviews that include the use of nanotechnology in various fields of dentistry, few of them comprehensively cover all applications in restorative dentistry. Therefore, the primary goal of this review is to draw attention to the multiple applications of nanomaterials in restorative dentistry. The review is divided into two parts. The first part was published in 2024, which addressed applications for remineralization inhibition and remineralization; the review stated that the nanoparticles (NPs) used are various and categorized into inorganic and organic NPs and concluded that they are very promising and provide insights into the practical use in the field of restorative dentistry [5]. This current review is the second part aimed at focusing on the reinforcement of restorative materials and other therapeutic applications of nanomaterials.

## Review

A comprehensive online search was performed in peer-reviewed publications, covering MEDLINE (Ovid), PubMed, Web of Science, Scopus, and Google Scholar databases to identify studies on the reinforcing and therapeutic applications of nanomaterials in restorative dentistry. The searches employed both controlled vocabulary and free-text phrases, as detailed in Table 1. This review addressed publications that met the following inclusion criteria: sourced from peer-reviewed journals indexed in the aforementioned database and published in English between 2014 and 2024. The articles were screened by the author by reviewing the abstracts and titles of all articles identified through computerized searches. Articles that definitely did not satisfy the inclusion criteria were excluded. All remaining articles were meticulously examined to ascertain adherence to the inclusion criteria.

**Table 1:** Literature search term components

Search terms						
Component 1	AND	Component 2	AND	Component 3	AND	Component 4
Nanofillers OR Nanomaterials OR Nanoparticles OR Nanotechnology		Restorative materials OR Restorative dentistry OR Conservative dentistry OR Dentistry		Materials reinforcement OR Mechanical properties OR Reinforced nanoparticles		Therapeutic OR Protective OR Drug delivery OR Nanorobots OR Major tooth repairs

In the previous review article (part I), which was published in 2024 [5], many aspects of nanotechnology were mentioned including nanomaterial fabrication and properties, classification of nanomaterials in restorative dentistry, overview of the application of nanomaterials in restorative dentistry, and in detail its application to enhance antimicrobial, anticaries, demineralization inhibition, and remineralization initiation properties as shown in Table 2. This current review (part II) will focus on the usage of NPs in restorative dentistry, either to improve the mechanical properties of the restorative materials or for diverse therapeutic applications, which is summarized in Table 3.

**Table 2:** Summary of demineralization inhibition and remineralization applications of nanomaterials in restorative dentistry

Nanofillers with antimicrobial, anticaries, and demineralization inhibition properties	Nanofillers to enhance remineralization
Silver nanoparticles	Metal nanoparticles
Zinc oxide nanoparticles	Nanohydroxyapatite
Titanium dioxide nanoparticles	Nano-amorphous calcium phosphate
Gold nanoparticles	Dicalcium phosphate nanoparticles
Silica nanoparticles	Calcium fluoride nanoparticles
Quaternary ammonium salt monomers	
Chitosan nanoparticles	

**Table 3:** Summary of reinforcement and therapeutic applications of nanomaterials in restorative dentistry

Nanofillers to reinforce restorative materials	Therapeutic miscellaneous applications	Nanorobot applications
Nanocarbon	Hypersensitivity management	Inducing anesthesia
Silica nanoparticles	Protective varnish	Major tooth repairs
Zirconia nanoparticles	Whitening effect	Dentition renaturalization
Hydroxyapatite nanoparticles	Drug delivery	Managing dentin hypersensitivity
Titanium dioxide nanoparticles	Nanoneedles	Tooth repositioning
Alumina nanoparticles	Nanorobots	Dentifrobots
Gold nanoparticles		Cancer diagnosis and therapy

NPs for reinforcement

Nanoscale particles have been observed to enhance the fracture toughness of nanocomposites in a pair of ways. The high surface-to-volume ratio promotes strong bonding at the interface, resulting in improved strength. Moreover, the exceptional durability of the nanosized particles helps to prevent particle breakup when interface cracking occurs. By utilizing nanosized particles, it is possible to enhance the interface toughness to elevated levels without the potential for particle fracture, hence enabling a greater fracture toughness value. NPs that are used for the reinforcement of restorative materials are nanocarbon, zirconia, hydroxyapatite, titanium dioxide, alumina, and gold [6].

Nanocarbon (Carbon Nanotubes)

Over the last 20 years, extensive research has focused on the advancement and utilization of carbon nanotubes (CNTs) in dentistry, mostly because of their exceptional mechanical capabilities, among other characteristics. They are very appropriate options for enhancing dental materials, serving as scaffolds, and improving targeted drug delivery systems [6].

CNTs are long, thin carbon cylinders formed by folding graphite sheets into a cylindrical shape. They are the most often utilized type of nanotubes in the dentistry field. Different types of nanotubes have been investigated for dental applications in several delightful fields. Nanotubes are available in either single-walled or multi-walled construction. It possesses outstanding mechanical characteristics in terms of rigidity, durability, and resilience, as well as exceptional bioactive capability. Single-walled CNTs (SWCNTs) possess exceptional mechanical characteristics, including a tensile strength ranging from 50 to 100 GPa and Young's modulus ranging from 1 to 2 TPa. These values are 5-10 times higher than those of steel, although the weight of SWCNTs is only 1/6th that of steel [7].

The main obstacle in using CNTs is the maximum amount of CNTs that can be included as a filler or reinforcement in polymers or other materials. This limitation arises due to the agglomeration of CNTs. Overcoming the challenge of CNT utilization often involves chemical functionalization to enhance their dispersion and reduce aggregation. Another problem is the color of CNTs, which is grayish, and when added to resin-based composites, it ruins the esthetics [6].

The benefits of adding CNTs to epoxy adhesives are enhancement in the mechanical strength and toughness of the bonded joints, a notable increase in the joints' adhesive bond strength, and enhancement in the water resistance of the adhesive joints as a result of the hydrophobic nature of CNTs. The addition of 1wt% and 5wt% multi-walled CNTs (MWCNTs) to the epoxy system resulted in an increase in shear strength of 31.2% and 45.6%, respectively [8].

The utilization of CNTs in reinforcing the dental composite has been observed to significantly improve its flexural strength and modulus of elasticity. This enhancement is achieved by reducing polymerization shrinkage and water resorption, resulting in the production of more durable and superior tooth restoratives. Also, carbon nanofibers have been used to reinforce dental adhesives, but they reinforced them to a lesser extent compared to the nanotubes. The predicted mechanical characteristics of carbon nanofibers are inferior to those exhibited by CNTs. Even so, nanofibers often possess longer lengths and are more cost-effective compared to CNTs [9].

Graphene is a carbon-based nanomaterial that is two-dimensional (2D). It is the thinnest and strongest element among various nanomaterials. Graphene-based materials can be classified into four groups: single-layer graphene, few-layered graphene, graphene oxide (GO), and reduced GO (rGO). Graphene and its derivatives have attracted considerable attention in the domains of medicine, biomedical research, and dentistry due to their amazing physical qualities, high electrical conductivity, and excellent biocompatibility. The applications involve tissue engineering, dental implant coatings, bone cement, resin additives, and tooth whitening. Graphene nanoplatelets (GNPs) are commonly utilized as additives in polymer dental adhesives due to their antibacterial and antibiofilm properties. The nanocomposites containing GNPs have demonstrated efficient inhibition of *Streptococcus mutans* cell activity while maintaining intact bonding characteristics [9].

Silica NPs

Scientists used nanotechnology to polish enamel to such a point that plaque could no longer adhere to it using techniques to polish enamel down to nanoscale roughness by using silica NPs (Si NPs) as a prophylaxis paste. The impact of Si NPs on the mechanical characteristics of dental resin composites was investigated by a combination of dynamic nanoindentation tests and macroscale tensile testing, confirming their role in enhancing mechanical strength [10]. Furthermore, the inclusion of silica fillers containing nanopores has been demonstrated to enhance the durability of posterior composite restorations. The presence of nanosized pores allows for the passage of monomers into and out of the fillers, hence enhancing the strength of the composite material [7].

Zirconia NPs

Zirconia NPs (ZrO_2_ NPs) have high strength and hardness properties; therefore, their addition would improve mechanical properties. They are also radiopaque particles, which would help improve radiographic diagnosis [11,12]. A study assessed the impact of conditioning with the phosphate ester monomer 10-methacryloyloxydecyl dihydrogen phosphate (MDP), with or without the application of zirconium hydroxide coating, on the mechanical properties of dental resin composites. The research revealed that the mechanical properties of resin composites are improved when nanosized zirconia fillers are treated with MDP, either with or without precoating with Zr(OH)_4_, and suggests that they can be safely used in clinical settings [13].

Hydroxyapatite NPs

A study was conducted to investigate the impact of incorporating calcium hydroxyapatite NPs (HA NPs) on the polymerization process and shear bond strength of Heliosit adhesive (Ivoclar Vivadent, Schaan, Liechtenstein). The results demonstrated a considerable variation in the degree of conversion and shear bond strength among all the groups examined. The group with an NP concentration of 2% by weight had the greatest values for both variables, while the group with an NP concentration of 4% by weight had the lowest value compared to the control group [14]. This proves the benefits of the incorporation of calcium HA NP into dental adhesive resins. The bond strength of resin-modified glass ionomers (RMGIs) was increased with the addition of Nano-HA (100-150 nm). The greater surface area available for bonding to the tooth structure when Nano-HA is used is probably what causes the improved strength. Nano-HA's larger surface area enhances its solubility and surface polish, making it easier to fill the tooth structure's demineralizing micropores. Enhanced mechanical properties, including compressive strength, diametral tensile strength, and biaxial flexural strength, were demonstrated by the nanofilled glass ionomers [14].

Titanium dioxide NPs

A study conducted a comparison and evaluation of the mechanical properties of an experimental composite resin containing 2.5% titanium dioxide NPs (TiO_2_ NPs) as a filler, in comparison to everX Flow (GC, Leuven, Belgium) and MultiCore Flow (Ivoclar Vivadent, Schaan, Liechtenstein). In comparison to the other materials that were examined, the experimental composite resin exhibited considerably higher values of compressive strength, diametral tensile strength, and flexural strength [15]. Another research examined how the shape of titanium dioxide nanofillers affects the dental composite's flexural strength and shear bond strength. Spherical and rhombic-shaped nano-titanium dioxide fillers were produced using the solvothermal process and then analyzed. These fillers were then added to a flowable composite (Filtek™ Z350 XT Flowable Restorative (3M ESPE, Dental Products, Saint Paul, MN, US)) at concentrations of 0.5wt% and 1.5wt%. This investigation validated the enhancing capability of titanium dioxide nanofillers on dental composite. However, the impact of utilizing nanofillers with varying morphology was shown to be insignificant [16].

Alumina NPs

The incorporation of alumina NPs (AL_2_O_3_ NPs) into the adhesive system leads to a significant enhancement in adhesive strength, with the greatest value achieved at a nanofiller content of 2wt%. The tensile strength of this adhesive, which has been strengthened with NPs, is approximately five times greater than the amount of a pure epoxy adhesive. The increase is directly connected to a change in the way failures occur. Specifically, the failures in non-modified adhesives are primarily interfacial, whereas in junctions bonded with nanoreinforced adhesives, the failures are a combination of cohesive and interfacial. When the amount of nanoalumina is high, the adhesive strength decreases due to the decrease in the adhesive's capacity for wetting the surface caused by the increase in its viscosity [8].

Gold NPs

Due to their significantly smaller dimensions than dentinal tubules, gold NPs (Au NPs) can enter the tubules more easily and with less resistance. The characteristic size-dependent pattern of Au NPs is one of its unique attributes at the nanoscale. When the NPs reach a diameter of around 5 nm, the melting temperature drops significantly from the value found in bulk gold. In conclusion, Au NPs may have a melting point that is less than 50% that of solid gold. Flexure, tensile strength, and resistance to displacement force were all improved when Au NPs were added to dental adhesive; this effect was directly correlated with the concentration of Au NPs [17].

Although of all the aforementioned applications of NPs for reinforcement of restorative materials, there is a need for additional statistical reviews to verify strength improvements through standardized comparison metrics for summarizing the mechanical properties of nanomaterials.

NPs for therapeutic applications

The great development in nanotechnology and its strong entry into all fields of science, medicine, and dentistry have contributed to opening broad horizons to provide modern and innovative methods for treating many dental patient problems with the utmost quality and with few complications. Among these promising applications are hypersensitivity management, protective varnish, whitening effect, drug delivery, nanoneedles, and nanorobots [3,4].

Hypersensitivity Management

Research has demonstrated that, in comparison to non-sensitive dentine, sensitive teeth have more dentinal tubules (35.6% versus 9.3%) and a larger diameter (0.83 µm against 0.43 µm). The tubules can be blocked by using Au NPs, which are the finest gold fillings available. Through the mechanism of photothermal conversion, Au NPs fused when exposed to laser irradiation [18].

Au NPs demonstrate a notable size-dependent phenomenon: as their size decreases, the melting point of the NPs decreases as well. The discrepancy of the melting temperature with the bulk value becomes notable as the size reaches approximately 5 nm in diameter. Two approaches were conducted to show the practicality of blocking exposed dentinal tubule holes using Au NPs. First, Au NPs are used to adhere to the inner walls of the tubules; then, silver staining is used to block the open dentinal tubules thereby decreasing hypersensitivity. Second, exposed open ends of dentinal tubules were brushed with highly concentrated Au NPs. This was followed by laser irradiation, which caused the Au NPs to photofuse by photothermal conversion. Au NPs possess high light absorption coefficients, resulting in the discovery of a remarkably efficient photothermal conversion effect. Consequently, they have been utilized in biomedical molecular imaging and therapeutic applications. The reason why Au NPs undergo photofusion is because they absorb light and then release that energy as heat to nearby particles, which is termed the photofusion phenomenon. In view of the high photothermal conversion effects of Au NPs and the consequent laser sintering process, it is probable to investigate potential strategies for dentinal tubule occlusion [18].

The use of a dentifrice containing 20% nanosized carbonate apatite is very effective in occluding the dentinal tubules. A randomized controlled trial studied the efficacy of dentifrice containing nano-carbonate apatite (n-CAP) and found that it effectively reduces dentine hypersensitivity (DH) following non-surgical periodontal therapy; the trial indicates that the use of n-CAP in the dentifrice could potentially reduce DH after four weeks of at-home treatment, in comparison to the control dentifrice [19,20].

Protective Varnish

An experimental acetone-based varnish containing nanofillers was prepared and applied on exposed dentin; the acetone solvent was evaporated through air-drying, while the curing process was carried out using a dental curing lamp that emits visible light for a duration of 20 seconds; and the Barcol hardness test and an abrasion resistance test were performed. The addition of nanofiller glass to the varnish ingredients significantly enhances its hardness and the abrasion resistance of the cured polymer. A novel varnish, made with 20% nanohydroxyapatite (Nano-HA) combined with 5% stannous chloride (SnCl_2_), was investigated for its effectiveness in reducing erosive-abrasive wear on bovine dentin. The results demonstrated that this varnish successfully blocked dentinal tubules and exhibited great potential as a preventive measure against erosive-abrasive wear in dentin [21].

Whitening Effect

Nanowhitening toothpaste is specifically designed for individuals with sensitive teeth and gums. It provides an effective whitening effect by utilizing calcium peroxide NPs (Nanoxyd). The toothpaste cleans teeth in a gentle manner using natural enzymes and has a very low level of abrasiveness. Additionally, it contains vitamin E, which promotes cell growth and revitalizes the gum [22].

The whitening properties of the newly developed Nano-HA toothpaste surpass those of commercially available whitening toothpaste. Nano-HA crystals have the ability to enter these small openings and function as a substance that lightens the color. This characteristic is a result of their enhanced surface area, which enables them to effectively remove stains from dental surfaces. Additionally, they fill the pores on the tooth surface and brighten its color. Currently, TiO_2_ NPs serve as agents for whitening. The utilization of nanotoothbrushes, along with the application of colloidal silver or gold between the bristles, serves as an effective measure to prevent the occurrence of gingivitis, periodontal disease, and tooth cavities. This technology significantly improves the elimination of microbial plaque [22].

Drug Delivery

Our objective for the future is to enhance the NPs' functionality and enable their control through external signals or local environmental factors. This could provide enhanced drug properties such as solubility, rate of dissolution, oral bioavailability, and targeting ability. Also, this would provide enhanced dosing requirements such as lower doses administered and better side effect profiles [23]. Nanodiamonds (NDs) have recently emerged in the field of medicine and dentistry. Their potential is currently being evaluated for utilization in many therapeutic approaches across multiple medical domains, including drug administration, tissue regeneration, and gene therapy. Recent research indicates that NDs could serve as bioactive or antibacterial coatings for dental implants [1,24].

Nanotubes have a wide range of applications in restorative dentistry, particularly in the field of controlled drug release. They can be utilized to chemically bind drugs to their surface by utilizing the chemical structure of nanotubes. Additionally, nanotubes are often used for carrying molecules within their lumen. Nanotubes have been utilized in the field of restorative dental materials primarily for the purpose of enhancing bioactivity, improving physicochemical characteristics, or serving as carriers for antimicrobial substances. Zeolites, metal-organic frameworks (MOFs), and nanostructured carbons are the primary families of porous nanomaterials. These materials are utilized to create functional materials that have significant applications in energy, environment, and medicine. Porous nanomaterials can serve as carriers for the cytotoxic agent that targets cancer cells. The drug can either be encapsulated within the pores and released upon reaching the cancer cell or be chemically linked to the material's surface by functionalization [25,26].

Although nanomaterials are revolutionizing the medical field, their potential applications in dentistry have yet to be fully explored. Dentistry is expected to undergo significant development in the coming years, as nanomaterials have the potential to modify current protocols for drug delivery and tissue engineering systems in the field of dentistry [24].

Nanoneedles

Novel suture needles containing nanoscale stainless steel crystals have been created. Researchers are currently working on the development of nanotweezers, which will enable cell surgery to be performed in a few years [3].

Nanorobots

A nanorobot is a highly specialized nanomachine with dimensions on the nanometer scale, often ranging from 0.1 to 10 microns. It consists of pieces that are chemically inert forms of carbon, measuring 1-100 nm in size. Medical nanorobots that are efficient may necessitate the inclusion of onboard computers to enable physicians to adequately monitor and regulate their operations. These programmable nanorobotic devices would allow precise intervention at the cellular and molecular levels; these are utilized to preserve and protect the human body from pathogens [27,28].

The applications of nanorobots in dentistry include inducing anesthesia, performing major tooth repairs, conducting renaturalization procedures, managing dentin hypersensitivity, facilitating tooth repositioning, utilizing dentifrobots, and employing nanorobots in cancer diagnosis and therapy [27].

Nanorobots for inducing anesthesia: The main concept is that nanotechnology employs several operative analgesic dental nanorobots, with dimensions in the nanometer range, which are suspended in a colloidal solution and used for local anesthesia. Upon reaching the dentin, nanorobots are capable of entering dentinal tubules, which have a diameter ranging from 1 to 4 μm, within a time frame of 100 seconds. Nanorobots move toward the pulp of the tooth, tracking chemical gradients, temperature variations, and navigational clues, all regulated by the nanocomputer on board and directed by the dentist. Once the therapy is finished, the dentist instructs the nanorobots to regain all sensation, give up control of nerve activity, and exit the tooth along the same pathway they entered. The benefits of nanorobots include enhanced patient comfort, decreased anxiety, needle-free administration, improved selectivity, precise control over the analgesic impact, rapid and fully reversible action, and the potential to prevent most side effects and complications [29].

Nanorobots for major tooth repair/nano-tissue engineering: The advancement of nanodental techniques for extensive tooth repair may progress through numerous stages of technical advancement, leading to genetic engineering, tissue engineering, tissue regeneration, and the growth of entirely new teeth in a controlled environment, followed by their implantation. The process of nanorobotic fabrication and implantation of a complete biological tooth, consisting of both mineral and cellular elements, was carried out in the field of nanotechnology. The objective of this procedure was to replicate the natural biomineralization process with the purpose of producing tooth enamel, which is the hardest tissue found in the human body. It involved the use of highly organized microarchitectural units called nanorods, which were composed of calcium hydroxyapatite crystals arranged in a roughly parallel manner [30].

Nanorobots for renaturalization procedures: Dentition renaturalization processes offer effective techniques for restoring partially damaged teeth using native biological components, eliminating the need for external filling materials. Full coronal renaturalizations will eliminate the need for fillings, crowns, and other dental restorations to repair damaged teeth [22].

Nanorobots for dentin hypersensitivity: Reconstructive dental nanorobots, utilizing natural biological substances, have the ability to selectively and accurately block specific dentinal tubules within a matter of minutes, providing patients with fast and permanent therapy [22].

Nanorobots for tooth repositioning: Orthodontic nanorobots have the ability to directly control the periodontal tissues, including the gingiva, periodontal ligament, cementum, and alveolar bone. This enables them to straighten, rotate, and vertically relocate teeth within a matter of minutes to hours in a quick and painless process [22].

Nanorobotic dentifrices (dentifrobots): Nanorobots designed for subocclusal residence can be administered through mouthwash or toothpaste. These nanorobots are capable of monitoring both supragingival and subgingival surfaces on a daily basis. They metabolize trapped organic debris, converting it into harmless and odorless fumes, while also continuously removing calculus. The proposed dentifrobots are extremely small, measuring between 1 and 10 microns in size, and move at a speed of 1-10 microns per second. They would be affordable and entirely mechanical in nature. These devices would have the ability to disable themselves if accidentally eaten and would be programmed with a precise occlusal avoidance protocol [31].

Nanorobots in cancer detection and treatment: Nanorobots can be utilized to detect and treat cancer. Nanorobots possess the ability to move within the bloodstream and can be utilized to address crucial areas of cancer therapy. The drawbacks of nanorobots are their expensive initial design cost, technical complexity, and susceptibility of electrical nanorobots to external electrical interference [32,33].

## Conclusions

Researchers have utilized nanotechnology to improve the physical and mechanical properties of existing materials and develop novel materials. Among the NPs that are used to reinforce the restorative materials are carbon, zirconia, hydroxyapatite, titanium dioxide, alumina, and gold. In the near future, nanodentistry may achieve optimal oral health by utilizing nanorobotics, nanomaterials, and biotechnology to enhance dental treatment through promising applications such as hypersensitivity management, protective varnish, whitening effect, and drug delivery, reaching into the complete regeneration of the periodontal apparatus, including dentine, cementum, periodontal ligaments, and bone, although there are still some limitations that must be addressed in the future.
